# Annotations

**Published:** 1909-03-13

**Authors:** 


### Annotations.

### The Late Editor of "The Lancet."

We have to record this week with much regret the death of one who bore a name intimately and honourably associated with medical journalism; and who himself devoted the greater part of his life to the expression and direction of medical opinion in the columns of the professional journal which owes its origin and most of its reputation and influence to successive members of the Wakley family. Only a comparatively short time ago we announced the passing away at a ripe old age of Thomas Henry Wakley, F.R.C.S.—father of the subject of this obituary notice, and son of the late Thomas Wakley, M.P., Coroner, editor, publicist, and medical reformer. It is now nearly ninety years ago since the first of the three Wakleys launched the *Lancet*, and with remarkable vigour, courage, and ability steered it through a stormy youth, and set it upon the steady and responsible course which has since been maintained. The third Thomas Wakley worthily carried on the traditions bound up with the *Lancet* and the family name, but was unhappily not destined for long life. His death on March 5, at his home in London, at the age of fifty-seven, is a severe loss to his paper, and to the medical world in general. Mr. Wakley was not one to obtrude his personality into public affairs, whether those of his own profession or of the world outside, and the interest he took in medical politics and the influence he wielded therein were both alike untouched by egotism or self-assertion. His early years were passed at Westminster School, whence he proceeded to Trinity College, Cambridge; and he subsequently studied medicine at St. Thomas's Hospital. After qualifying in 1883 he joined his father upon the editorial staff of the family paper. The *Lancet* was Thomas Wakley's life-work: he was born and bred to it, and could scarcely have done otherwise than follow where his father and grandfather had led the way. If his personality was less striking than theirs, its quiet steady influence was directed towards the same ends, and with a like result; and Medicine and Journalism as well as a wide circle of friends are the poorer for his death.

### The Opium Commission.

The International Opium Commission, which has been sitting in Shanghai, has concluded its deliberations, and the resolutions adopted have in substance transpired. The Commission recognises that the Chinese Government is sincere in its desire to suppress opium smoking—a point which has been often called in question by those who fancied that the real anxiety was to promote the prosperity of native agriculturists by excluding the Indian-grown crops from the markets. It also finds that the morphine habit shows signs of spreading, and constitutes already a very grave danger. Of the other findings but two have much interest from a medical standpoint, and they deal with anti-opium remedies and the properties and effects of opium itself and of its derivatives. On both these points the Commission formulates no conclusion, but is content to recommend the subject to the various Governments concerned for such action as may be thought necessary. Now in shelving a part of the whole opium problem in this way the Commission has missed a great opportunity of settling a quasi-medical, quasi-ethnological point which is of interest and importance. It has been alleged, by observers both numerous and competent, that, by some subtle fundamental constitutional difference, Eastern races are able to indulge in opium without the necessity of constantly increasing the dose required to produce soothing and narcotic effects. And it has been argued from this that to repress opium consumption in China with a heavy hand is as unjustifiable as to enforce total abstinence upon the moderate drinker in Great Britain. The Chinese Government, it is clear, does not adopt this attitude; but the recent Commission could, had it chosen, have settled this point once and for all by a scientific investigation, and it seems a pity that no effort in that direction was ordered.

### The Middlesex Hospital Cancer Charity.

If good wishes could speed the cause of cancer research, or provide the sinews of the war necessary for its prosecution, the authorities of the Middlesex Hospital and its cancer charity are well off indeed. The very best of the journalistic talent of the daily Press in this country seems to be on their side, and the comments which have appeared since the annual meeting of the Governors of the hospital on February 25 must be highly gratifying to all concerned, whether laboratory workers, clinicians, or managerial authorities. This is the more admirable in that, to those with no technical knowledge of the great intricacy of the lines of research which are being followed out, the progress of the work seems beyond question slow to the verge of disheartenment. At the same time, even the tyro in scientific matters must be impressed with the volume of work which has already been achieved, and the definite, if often negative, results which have been attained, by reading the voluminous annual reports issued by the Research Laboratories Department. In this connection attention has rightly been called to the fact that the Director, Dr. Lazarus-Barlow, has been invited to give the Croonian Lectures before the Royal College of Physicians in June, and that his subject will be the bearing of the researches for which he is responsible upon the problems of cancer. Furthermore, as Dr. Lazarus-Barlow pointed out in his report, a Hunterian Professorship has been held for four consecutive years by one member or another of the laboratory staff. He further gave a very happy illustration of the immensity of the work which lies before him and his fellow-workers by comparing their task with that of building a *Dreadnought* if the shipbuilders had to start by quarrying and smelting their iron ore, cutting their own timber, and in fact going back to the first principles and prime sources generally. We trust that as a result of all this excellent publicity the funds and work of the Cancer Research Department may materially benefit.

